# The genome of Roselle's flesh fly *Sarcophaga* ( *Helicophagella*) *rosellei* *(*Böttcher, 1912)

**DOI:** 10.12688/wellcomeopenres.18874.1

**Published:** 2023-01-31

**Authors:** Steven Falk, University of Oxford and Wytham Woods Genome Acquisition Lab, Darwin Tree of Life Barcoding collective, Wellcome Sanger Institute Tree of Life programme, Wellcome Sanger Institute Scientific Operations: DNA Pipelines collective, Tree of Life Core Informatics collective, John F. Mulley, Darwin Tree of Life Consortium

**Affiliations:** 1 Independent researcher, Kenilworth, Warwickshire, UK; 2 School of Natural Sciences, Bangor University, Bangor, Wales, UK

**Keywords:** Sarcophaga rosellei, Roselle’s flesh fly, genome sequence, chromosomal, Diptera

## Abstract

We present a genome assembly from an individual male *Sarcophaga rosellei* (Roselle's flesh fly; Arthropoda; Insecta; Diptera; Sarcophagidae). The genome sequence is 541 megabases in span. Most of the assembly is scaffolded into six chromosomal pseudomolecules, with the X sex chromosome assembled. The mitochondrial genome has also been assembled and is 19.5 kilobases in length. Gene annotation of this assembly on Ensembl has identified 15,437 protein coding genes.

## Species taxonomy

Eukaryota; Metazoa; Ecdysozoa; Arthropoda; Hexapoda; Insecta; Pterygota; Neoptera; Endopterygota; Diptera; Brachycera; Muscomorpha; Oestroidea; Sarcophagidae; *Sarcophaga*; *Helicophagella*; *Sarcophaga rosellei* ( Böttcher, 1912) (NCBI:txid1206372).

## Background

Roselle’s flesh fly ( *Sarcophaga rosellei*) is a medium-sized (6.5–11 mm) ( van Emden, 1954) flesh fly with a Palearctic distribution ( Pape, 1996). The species was named by Böttcher in 1912 in honour of Dr du Roselle, who produced the first illustrations of male Sarcophagid genitalia ( Böttcher, 1912; Senior-White, 1924). As with other members of the genus, *S. rosellei* has an overall grey/black colouration, with large red or orange eyes, three longitudinal stripes on the thorax, and a checked abdomen. *S. rosellei* is found across England and Wales, where it is most common from May to August, but is scarce in Scotland ( NBN Atlas, Accession number NBNSYS0000156291).

The genus *Sarcophaga* contains roughly 890 species divided into around 69 subgenera ( Buenaventura *et al.,* 2017), and *S. rosellei* is placed in the *Helicophagella* subgenus, along with four other UK Sarcophagid species ( *S. agnata; S. crassimargo*; *S. hirticrus*; *S. melanura*). *Helicophagella* is probably not monophyletic ( Buenaventura & Pape, 2017), and is split into two subgroups: the *noverca* group and the *melanura* group, roughly along dietary lines, with *melanura* group members breeding in faeces and *noverca* group members breeding in snails ( Blackith *et al.,* 1997). *S. rosellei* is a member of the noverca group and has been recorded as preying on snails, a relationship that may explain the association of *S. rosellei* with calcareous soils ( Blackith *et al.,* 1997; Rozkošný & Vanhara, 1993). The *S. rosellei* genome assembly, together with those of other *Sarcophaga* species from the Darwin Tree of Life Project and elsewhere, is likely to be of great benefit to resolving the phylogeny of the genus and identifying patterns of dietary shifts.

### Genome sequence report

**Figure 1.**
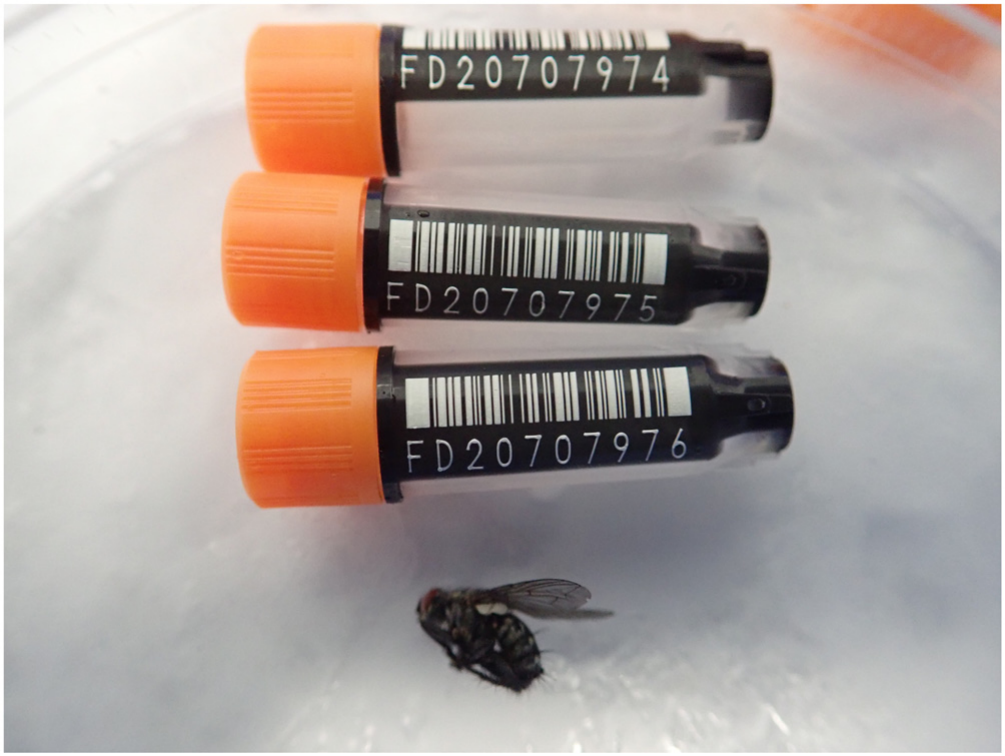
Image of the *Sarcophaga rosellei* (idSarRose1) specimen used for genome sequencing.

The genome was sequenced from one male *Sarcophaga rosellei* specimen ( Figure 1) collected from Wytham Woods, Oxfordshire (biological vice-county: Berkshire), UK (latitude 51.77, longitude –1.33). A total of 54-fold coverage in Pacific Biosciences single-molecule HiFi long reads and 65-fold coverage in 10X Genomics read clouds were generated. Primary assembly contigs were scaffolded with chromosome conformation Hi-C data. Manual assembly curation corrected 19 missing joins or mis-joins and removed three haplotypic duplications, reducing the scaffold number by 7.14%, and increasing the scaffold N50 by 2.23%.

**Figure 2.**
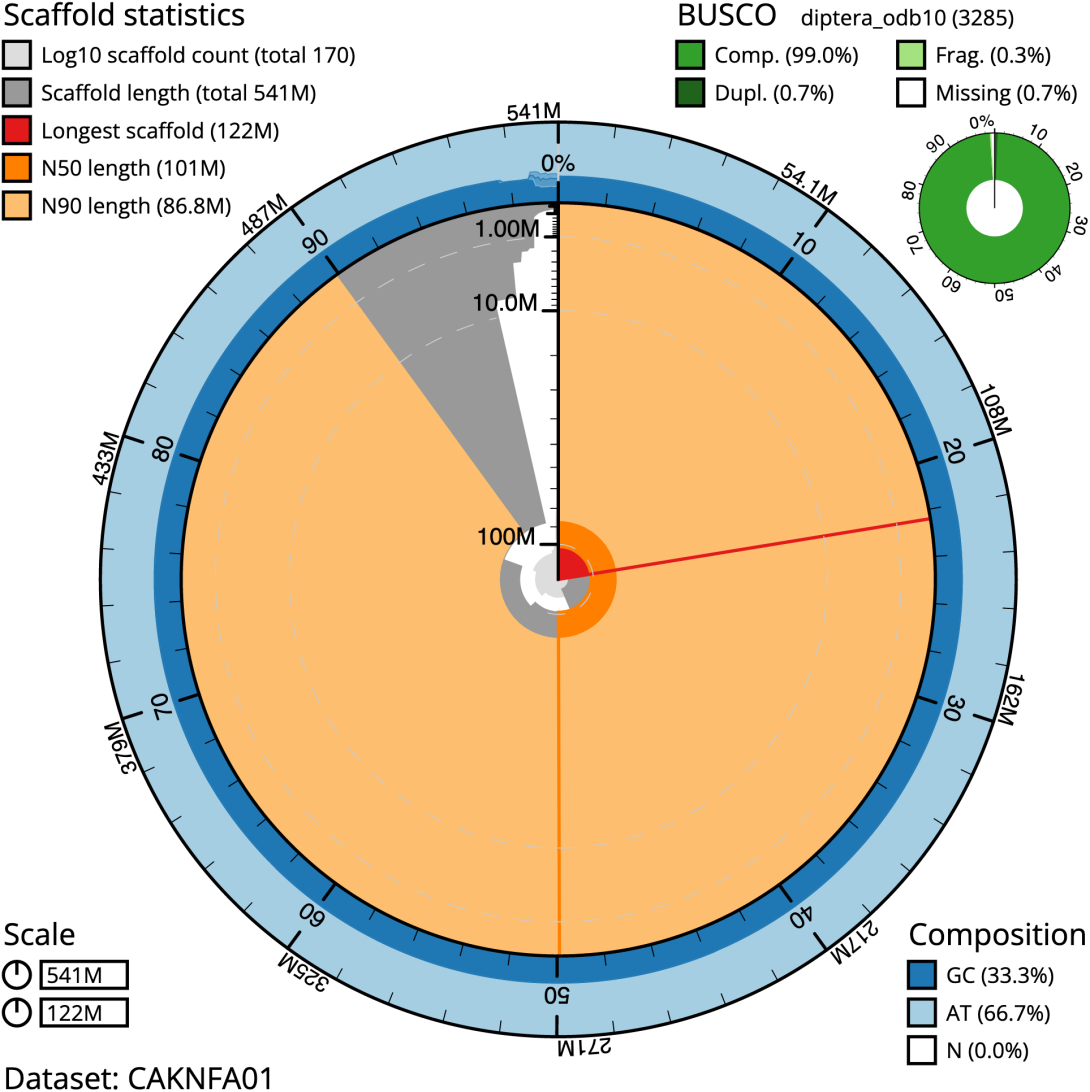
Genome assembly of *Sarcophaga rosellei*, idSarRose1.1: metrics. The BlobToolKit Snailplot shows N50 metrics and BUSCO gene completeness. The main plot is divided into 1,000 size-ordered bins around the circumference with each bin representing 0.1% of the 541,393,943 bp assembly. The distribution of scaffold lengths is shown in dark grey with the plot radius scaled to the longest scaffold present in the assembly (121,533,712 bp, shown in red). Orange and pale-orange arcs show the N50 and N90 scaffold lengths (101,196,005 and 86,778,848 bp), respectively. The pale grey spiral shows the cumulative scaffold count on a log scale with white scale lines showing successive orders of magnitude. The blue and pale-blue area around the outside of the plot shows the distribution of GC, AT and N percentages in the same bins as the inner plot. A summary of complete, fragmented, duplicated and missing BUSCO genes in the diptera_odb10 set is shown in the top right. An interactive version of this figure is available at https://blobtoolkit.genomehubs.org/view/idSarRose1.1/dataset/CAKNFA01/snail.

The final assembly has a total length of 541.4 Mb in 169 sequence scaffolds with a scaffold N50 of 101.2 Mb ( Table 1). Most (98.81%) of the assembly sequence was assigned to six chromosomal-level scaffolds, representing five autosomes and the X sex chromosome. Chromosome-scale scaffolds are named by synteny based on the genome assembly of *Sarcophaga caerulescens* GCA_927399465.1 ( Figure 2– Figure 5; Table 2). The assembly has a BUSCO v5.3.2 ( Manni *et al.*, 2021) completeness of 99.0% (single 98.3%, duplicated 0.7%) using the OrthoDB v10 Diptera reference set. While not fully phased, the assembly deposited is of one haplotype. Contigs corresponding to the second haplotype have also been deposited.

**Table 1.** Genome data for *Sarcophaga rosellei*, idSarRose1.1.

Project accession data		
Assembly identifier	idSarRose1.1	
Species	*Sarcophaga rosellei*	
Specimen	idSarRose1	
NCBI taxonomy ID	1206372	
BioProject	PRJEB47785	
BioSample ID	SAMEA7746603	
Isolate information	male idSarRose1 (thorax: 10X and PacBio; abdomen: RNA-Seq; head: Hi-C)	
Assembly metrics *		*Benchmark*
Consensus quality (QV)	57.7	*≥ 50*
*k*-mer completeness	100%	*≥ 95%*
BUSCO **	C:99.0%[S:98.3%,D:0.7%], F:0.3%,M:0.7%,n:3,285	*C ≥ 95%*
Percentage of assembly mapped to chromosomes	98.81%	*≥ 95%*
Sex chromosomes	X chromosome	*localised homologous pairs*
Organelles	Mitochondrial genome assembled	*complete single alleles*
**Raw data accessions**		
PacificBiosciences SEQUEL II	ERR7012654, ERR7015068	
10X Genomics Illumina	ERR6895901–ERR6895904	
Hi-C Illumina	ERR6895900	
PolyA RNA-Seq Illumina	ERR10123657	
**Genome assembly**		
Assembly accession	GCA_930367235.1	
*Accession of alternate haplotype*	GCA_930367195.1	
Span (Mb)	541.4	
Number of contigs	208	
Contig N50 length (Mb)	44.8	
Number of scaffolds	169	
Scaffold N50 length (Mb)	101.2	
Longest scaffold (Mb)	121.5	
**Genome annotation**		
Number of protein-coding genes	15,437	
Non-coding genes	9,457	
Number of transcripts	35,327	

* Assembly metric benchmarks are adapted from column VGP-2020 of “Table 1: Proposed standards and metrics for defining genome assembly quality” from ( Rhie *et al.*, 2021). ** BUSCO scores based on the diptera_odb10 BUSCO set using v5.3.2. C = complete [S = single copy, D = duplicated], F = fragmented, M = missing, n = number of orthologues in comparison. A full set of BUSCO scores is available at https://blobtoolkit.genomehubs.org/view/idSarRose1.1/dataset/CAKNFA01/busco.

**Figure 3.**
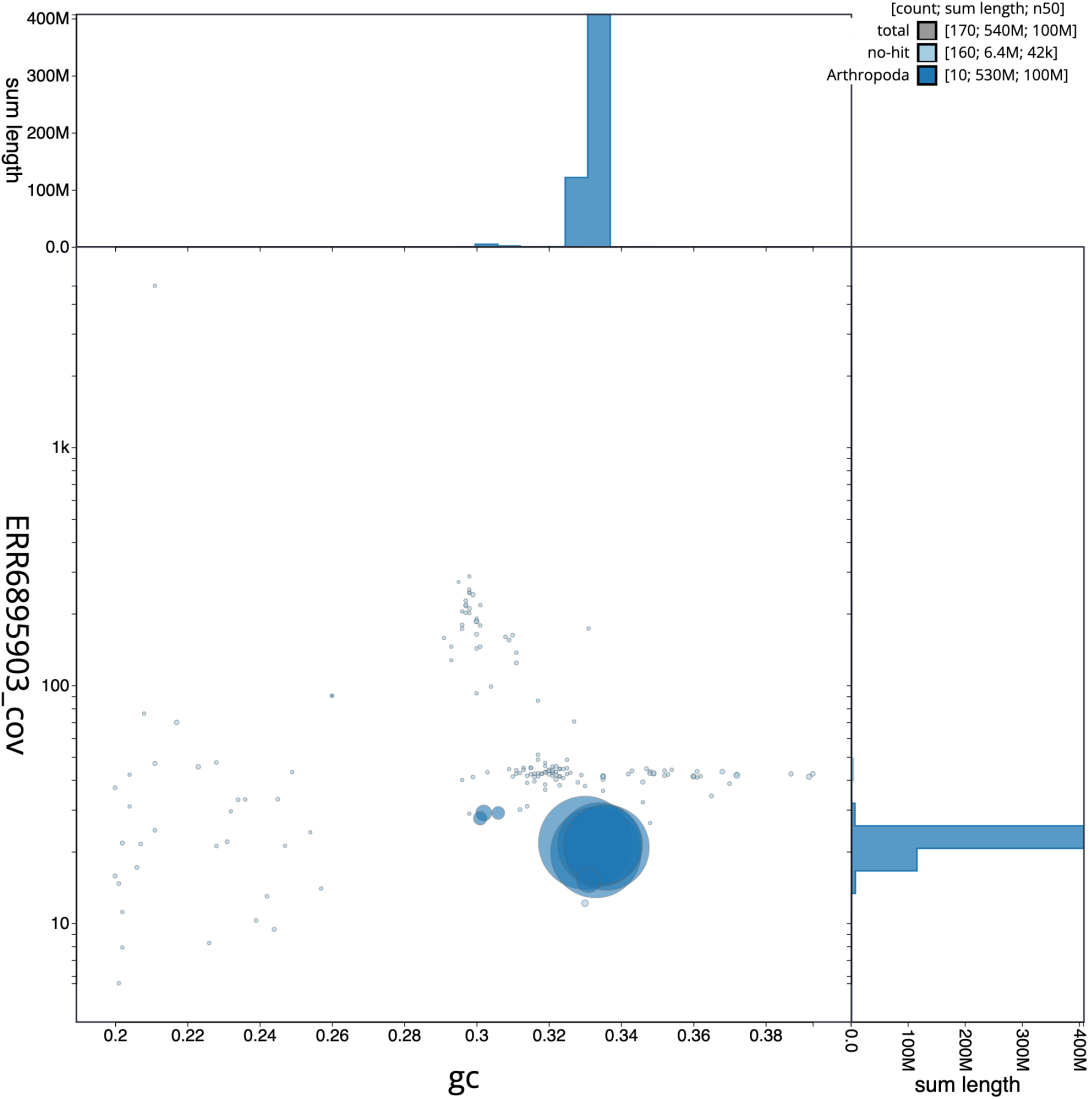
Genome assembly of *Sarcophaga rosellei*, idSarRose1.1: GC coverage. BlobToolKit GC-coverage plot. Scaffolds are coloured by phylum. Circles are sized in proportion to scaffold length. Histograms show the distribution of scaffold length sum along each axis. An interactive version of this figure is available at https://blobtoolkit.genomehubs.org/view/idSarRose1.1/dataset/CAKNFA01/blob.

**Figure 4.**
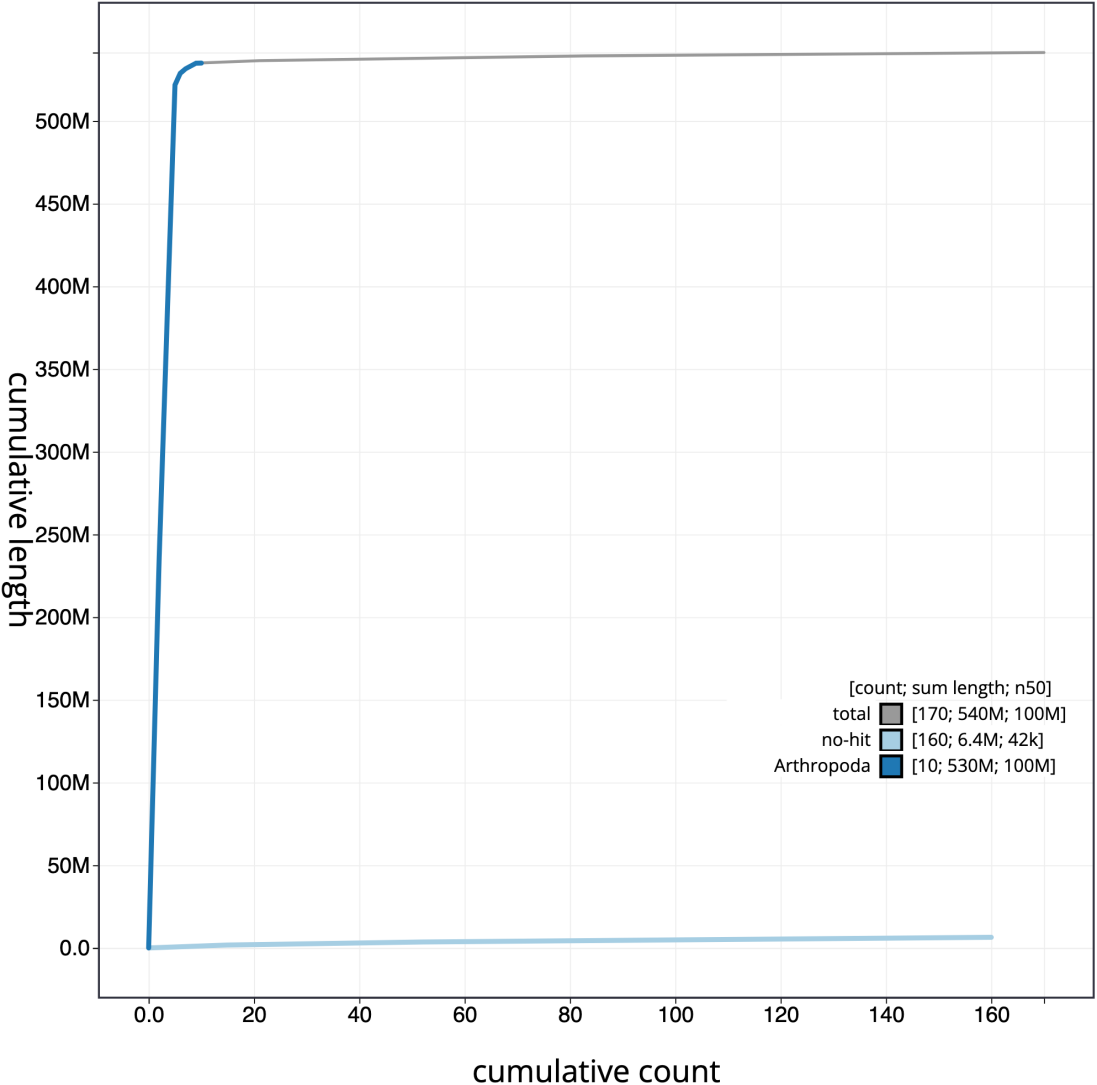
Genome assembly of *Sarcophaga rosellei*, idSarRose1.1: cumulative sequence. BlobToolKit cumulative sequence plot. The grey line shows cumulative length for all scaffolds. Coloured lines show cumulative lengths of scaffolds assigned to each phylum using the buscogenes taxrule. An interactive version of this figure is available at https://blobtoolkit.genomehubs.org/view/idSarRose1.1/dataset/CAKNFA01/cumulative.

**Figure 5.**
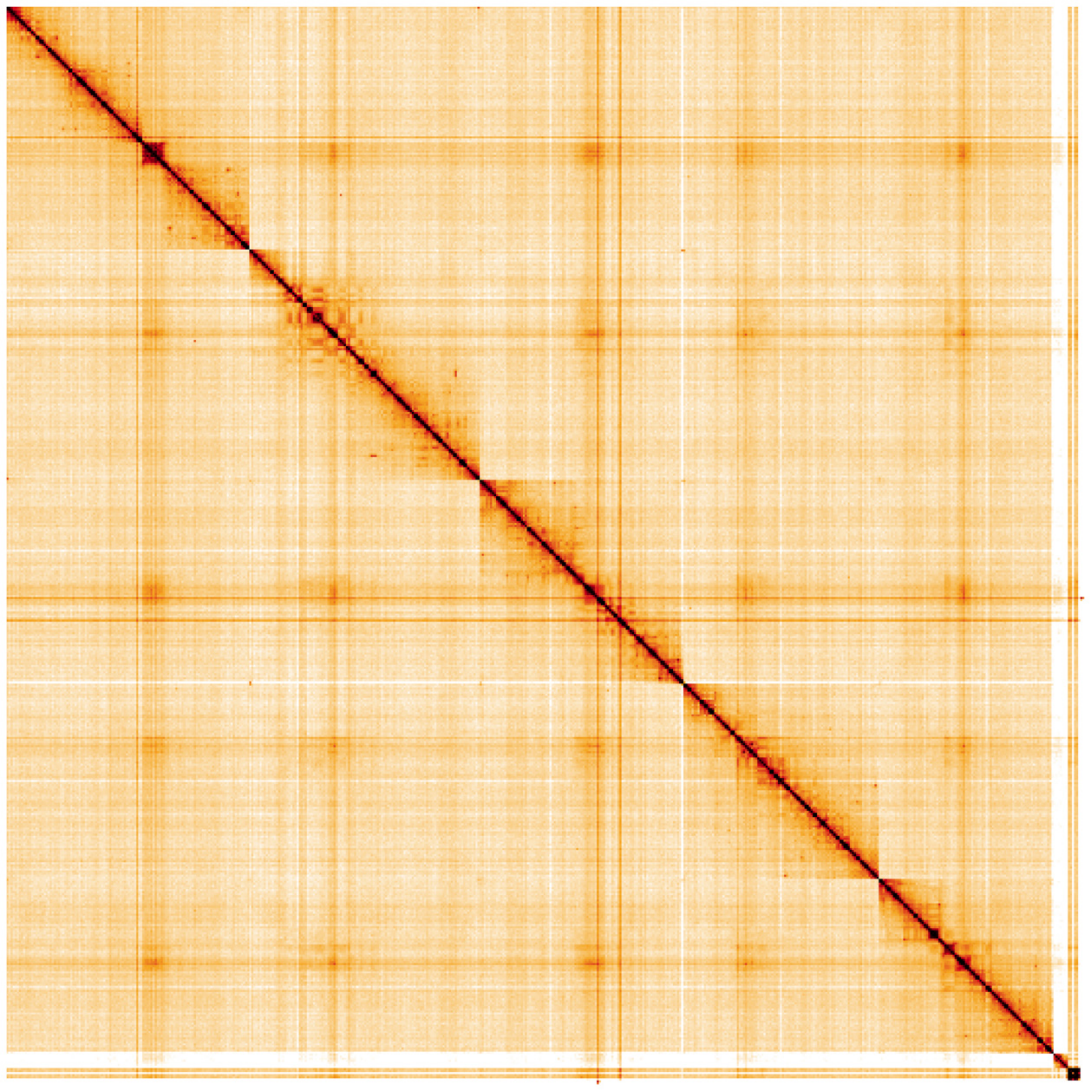
Genome assembly of *Sarcophaga rosellei*, idSarRose1.1: Hi-C contact map. Hi-C contact map of the idSarRose1.1 assembly, visualised using HiGlass. Chromosomes are shown in order of size from left to right and top to bottom. An interactive version of this figure may be viewed at https://genome-note-higlass.tol.sanger.ac.uk/l/?d=MIvbf0y8SPi6oXdSK5TiJA.

**Table 2.** Chromosomal pseudomolecules in the genome assembly of *Sarcophaga rosellei*, idSarRose1.

INSDC accession	Chromosome	Size (Mb)	GC%
OV884017.1	1	121.53	33
OV884018.1	2	114.72	33.4
OV884019.1	3	101.2	33.6
OV884020.1	4	97.58	33.4
OV884021.1	5	86.78	33.5
OV884022.1	X	7.04	33.1
OV884023.1	MT	0.02	21.1
-	unplaced	12.54	30.7

### Genome annotation report

The GCA_930367235.1 genome assembly was annotated using the Ensembl rapid annotation pipeline ( Table 1; https://rapid.ensembl.org/Sarcophaga_rosellei_GCA_930367235.1/). The resulting annotation includes 35,327 transcribed mRNAs from 15,437 protein-coding and 9,457 non-coding genes.

## Methods

### Sample acquisition and nucleic acid extraction

A male *Sarcophaga rosellei* specimen (idSarRose1) was collected using a net in Wytham Woods, Oxfordshire (biological vice-county: Berkshire), UK (latitude 51.77, longitude –1.33) on 4 August 2020. The specimen was collected and identified by Steven Falk (independent researcher). The specimen was snap-frozen on dry ice.

DNA was extracted at the Tree of Life laboratory, Wellcome Sanger Institute (WSI). The idSarRose1 sample was weighed and dissected on dry ice with tissue set aside for Hi-C sequencing. Thorax tissue was disrupted using a Nippi Powermasher fitted with a BioMasher pestle. High molecular weight (HMW) DNA was extracted using the Qiagen MagAttract HMW DNA extraction kit. Low molecular weight DNA was removed from a 20-ng aliquot of extracted DNA using 0.8X AMpure XP purification kit prior to 10X Chromium sequencing; a minimum of 50 ng DNA was submitted for 10X sequencing. HMW DNA was sheared into an average fragment size of 12–20 kb in a Megaruptor 3 system with speed setting 30. Sheared DNA was purified by solid-phase reversible immobilisation using AMPure PB beads with a 1.8X ratio of beads to sample to remove the shorter fragments and concentrate the DNA sample. The concentration of the sheared and purified DNA was assessed using a Nanodrop spectrophotometer and Qubit Fluorometer and Qubit dsDNA High Sensitivity Assay kit. Fragment size distribution was evaluated by running the sample on the FemtoPulse system.

RNA was extracted from abdomen tissue of idSarRose1 in the Tree of Life Laboratory at the WSI using TRIzol, according to the manufacturer’s instructions. RNA was eluted in 50 μL RNAse-free water and its concentration was assessed using a Nanodrop spectrophotometer and Qubit Fluorometer using the Qubit RNA Broad-Range (BR) Assay kit. Analysis of the integrity of the RNA was done using Agilent RNA 6000 Pico Kit and Eukaryotic Total RNA assay.

### Sequencing

Pacific Biosciences HiFi circular consensus and 10X Genomics read cloud DNA sequencing libraries were constructed according to the manufacturers’ instructions. Poly(A) RNA-Seq libraries were constructed using the NEB Ultra II RNA Library Prep kit. DNA and RNA sequencing was performed by the Scientific Operations core at the WSI on Pacific Biosciences SEQUEL II (HiFi), Illumina NovaSeq 6000 (RNA-Seq and 10X) instruments. Hi-C data were also generated from head tissue of idSarRose1 using the Arima v2 kit and sequenced on the Illumina NovaSeq 6000 instrument.

### Genome assembly

Assembly was carried out with Hifiasm ( Cheng *et al.*, 2021) and haplotypic duplication was identified and removed with purge_dups ( Guan *et al.*, 2020). One round of polishing was performed by aligning 10X Genomics read data to the assembly with Long Ranger ALIGN, calling variants with freebayes ( Garrison & Marth, 2012). The assembly was then scaffolded with Hi-C data ( Rao *et al.*, 2014) using SALSA2 ( Ghurye *et al.*, 2019). The assembly was checked for contamination as described previously ( Howe *et al.*, 2021). Manual curation was performed using HiGlass ( Kerpedjiev *et al.*, 2018) and Pretext ( Harry, 2022). The mitochondrial genome was assembled using MitoHiFi ( Uliano-Silva *et al.*, 2022), which performed annotation using MitoFinder ( Allio *et al.*, 2020). The genome was analysed and BUSCO scores were generated within the BlobToolKit environment ( Challis *et al.*, 2020). Table 3 contains a list of all software tool versions used, where appropriate.

**Table 3.** Software tools and versions used.

Software tool	Version	Source
BlobToolKit	3.4.0	Challis *et al.*, 2020
freebayes	1.3.1-17-gaa2ace8	Garrison & Marth, 2012
Hifiasm	0.15.3	Cheng *et al.*, 2021
HiGlass	1.11.6	Kerpedjiev *et al.*, 2018
Long Ranger ALIGN	2.2.2	https://support.10xgenomics.com/genome-exome/ software/pipelines/latest/advanced/other-pipelines
MitoHiFi	2	Uliano-Silva *et al.*, 2022
PretextView	0.2	Harry, 2022
purge_dups	1.2.3	Guan *et al.*, 2020
SALSA	2.2	Ghurye *et al.*, 2019

### Genome annotation

The Ensembl gene annotation system ( Aken *et al.*, 2016) was used to generate annotation for the *S. rosellei* assembly GCA_930367235.1. Annotation was created primarily through alignment of transcriptomic data to the genome, with gap filling via protein to-genome alignments of a select set of proteins from UniProt ( UniProt Consortium, 2019).

### Ethics/compliance issues

The materials that have contributed to this genome note have been supplied by a Darwin Tree of Life Partner. The submission of materials by a Darwin Tree of Life Partner is subject to the Darwin Tree of Life Project Sampling Code of Practice. By agreeing with and signing up to the Sampling Code of Practice, the Darwin Tree of Life Partner agrees they will meet the legal and ethical requirements and standards set out within this document in respect of all samples acquired for, and supplied to, the Darwin Tree of Life Project. Each transfer of samples is further undertaken according to a Research Collaboration Agreement or Material Transfer Agreement entered into by the Darwin Tree of Life Partner, Genome Research Limited (operating as the Wellcome Sanger Institute), and in some circumstances other Darwin Tree of Life collaborators.

## Data Availability

European Nucleotide Archive: *Sarcophaga rosellei*. Accession number PRJEB47785; https://identifiers.org/ena.embl/PRJEB47785. ( Wellcome Sanger Institute, 2021) The genome sequence is released openly for reuse. The *Sarcophaga rosellei* genome sequencing initiative is part of the Darwin Tree of Life (DToL) project. All raw sequence data and the assembly have been deposited in INSDC databases. Raw data and assembly accession identifiers are reported in Table 1.

## Author information

Members of the University of Oxford and Wytham Woods Genome Acquisition Lab are listed here: https://doi.org/10.5281/zenodo.4789928.

Members of the Darwin Tree of Life Barcoding collective are listed here: https://doi.org/10.5281/zenodo.4893703.

Members of the Wellcome Sanger Institute Tree of Life programme are listed here: https://doi.org/10.5281/zenodo.4783585.

Members of Wellcome Sanger Institute Scientific Operations: DNA Pipelines collective are listed here: https://doi.org/10.5281/zenodo.4790455.

Members of the Tree of Life Core Informatics collective are listed here: https://doi.org/10.5281/zenodo.5013541.

Members of the Darwin Tree of Life Consortium are listed here: https://doi.org/10.5281/zenodo.4783558.
