# Supplementary material for: The genome of Roselle's flesh fly Sarcophaga ( Helicophagella) rosellei (Böttcher, 1912)
Source: Wellcome Open Res. 2023 Jan 31;8:43. [Version 1] doi: 10.12688/wellcomeopenres.18874.1 (PMC10521103; doi:10.12688/wellcomeopenres.18874.1)
Supplement: Peer review history

# Reviewer report: Reviewer response for version 1

**DOI:** 10.21956/wellcomeopenres.20928.r65827

**Published:** 2023-09-08

**Authors:** Fan Song

**Affiliations:** 1 Department of Entomology and MOA Key Lab of Pest Monitoring and Green Management College of Plant Protection, China Agricultural University, Beijing, China

This article presented a genome assembly of Roselle's flesh fly *Sarcophaga rosellei *from an individual male adult. This manuscript is generally well-written and described this genome clearly. Two minor adjustments should be addressed. 

- It is suggested that the horizontal and vertical coordinates of Figure 5 could have a label showing the size of the genome and indicating which is the X chromosome.
- It is recommended to add annotation of repeat sequences of the genome.

Are sufficient details of methods and materials provided to allow replication by others?

Yes

Is the rationale for creating the dataset(s) clearly described?

Yes

Are the datasets clearly presented in a useable and accessible format?

Yes

Are the protocols appropriate and is the work technically sound?

Yes

Reviewer Expertise:

Insect Genome; Phylogenomics

I confirm that I have read this submission and believe that I have an appropriate level of expertise to confirm that it is of an acceptable scientific standard.

# Reviewer report: Reviewer response for version 1

**DOI:** 10.21956/wellcomeopenres.20928.r54589

**Published:** 2023-02-02

**Authors:** Thomas Pape

**Affiliations:** 1 Natural History Museum of Denmark, Science Faculty, University of Copenhagen, Copenhagen, Denmark

This is another very useful flesh fly genome published by Falk *et al*., and I am very happy to see this. My comments are mainly on the flesh-fly side of things and not on the technicalities of the assembly, but hopefully my input is still useful.

 Comments in the attached PDF.

Are sufficient details of methods and materials provided to allow replication by others?

Yes

Is the rationale for creating the dataset(s) clearly described?

Yes

Are the datasets clearly presented in a useable and accessible format?

Yes

Are the protocols appropriate and is the work technically sound?

Yes

Reviewer Expertise:

Taxonomy, phylogeny, systematics and evolution of calyptrate flies, including morphology, anatomy and ecology.

I confirm that I have read this submission and believe that I have an appropriate level of expertise to confirm that it is of an acceptable scientific standard.
